# Malaria and gravidity interact to modify maternal haemoglobin concentrations during pregnancy

**DOI:** 10.1186/1475-2875-11-348

**Published:** 2012-10-22

**Authors:** Smaïla Ouédraogo, Florence Bodeau-Livinec, Valérie Briand, Bich-Tram Huynh, Ghislain K Koura, Manfred MK Accrombessi, Nadine Fievet, Achille Massougbodji, Philippe Deloron, Michel Cot

**Affiliations:** 1 Mère et enfant face aux infections tropicales, IRD Unité mixte de recherche 216, Paris, France; 2 Ecole des Hautes Etudes en Santé Publique, Rennes, France; 3 Laboratoire de Parasitologie, Faculté des Sciences de la Santé, Cotonou, Benin; 4 PRES Sorbonne Paris Cité, Faculté de pharmacie, Paris, France

**Keywords:** Anaemia, Gravidity, Malaria, Iron deficiency, Prevention

## Abstract

**Background:**

Primigravidity is one of the main risk factors for both malaria and anaemia. Since the implementation of intermittent preventive treatment (IPTp) in sub-Saharan Africa, the relationship between anaemia and gravidity and its evolution during pregnancy has been little explored. This study aimed to evaluate the impact of gravidity on the variation of haemoglobin during pregnancy according to the timing of gestation.

**Methods:**

Data from three studies carried out in nearby areas in south Benin (Ouidah, Comé, Allada) between 2005 and 2012 were analysed. At inclusion (first antenatal visit, ANV1) women’s age, area of residence, schooling, gravidity, gestational age, weight and height were recorded. Thick blood smears were performed on ANV1, second visit (ANV2) and at delivery. In Allada, women’s serum ferritin and CRP concentrations were also assessed. The impact of gravidity on maternal haemoglobin (Hb) was analysed using a logistic or linear regression depending on the outcome. The statistical significance was set to P < 0.05.

**Results:**

In total, data from 3,591 pregnant women were analysed. Both univariate and multivariate analyses showed a constant association between Hb concentrations and gravidity in the three periods of Hb assessment (ANV1, ANV2 and delivery). Mean Hb concentration was significantly lower in primigravidae than in multigravidae at ANV1 (mean difference = -2.4 g/L, CI 95%: [-3.4, -1.4], P < 0.001). Afterwards, there was a significant increase in primigravidae only, with a tendency to reversal between primigravidae and multigravidae, which was confirmed at delivery (mean difference = 2.8 g/L, CI 95%: [1.3, 4.2], P < 0.001). The prevalence of malaria infection was halved between ANV1 and delivery in primigravidae while it decreased by only 38% among multigravidae, who were less prone to malaria infection (prevalence at ANV1, 20% and 10% respectively). Iron deficiency was more common in multigravidae, and it decreased slightly in this group between ANV1 and delivery.

**Conclusion:**

In a context of IPTp, Hb levels improved progressively throughout pregnancy in primigravidae, likely as a result of reduction in malaria infection. In multigravidae, the improvement was less perceptible and anaemia was mainly due to iron deficiency.

## Background

As part of the prospective cohort study (Anaemia in Pregnancy: Etiologies and Consequences “APEC”) carried out in Benin between 2010 and 2012, primigravidae and multigravidae were followed from the first antenatal visit (ANV1) until delivery. In a preceding paper [1], analyses showed that the administration of anti-parasitic treatments (IPTp with SP or mefloquine and albendazole) at ANV1 significantly decreased the prevalence of malaria infection and helminths. Furthermore, these analyses showed that malaria and helminth infestations were no longer associated with anaemia at the second antenatal visit (ANV2). At ANV2, the effect of primigravidity on anaemia, particularly marked at enrolment, also disappeared, suggesting this group could be particularly sensitive to preventive measures.

First pregnancy is recognized as one of the main risk factors for both malaria and anaemia [2-4]. The variations of haemoglobin (Hb) levels throughout pregnancy have seldom been investigated, although an early study in Papua New Guinea, where pregnant women received neither malaria prophylaxis nor haematinics, reported that the differences in Hb levels between malaria-positive at booking and malaria-negative at delivery were higher in primigravidae than in multigravidae [5].

However, since the implementation of IPTp in sub-Saharan Africa, no clinical trial has studied the effect of malaria-focused preventive measures on anaemia in relation to gravidity. To evaluate the impact of gravidity on the variation of Hb during pregnancy according to the timing of gestation, data from APEC were analysed and further pooled with two earlier studies, which had followed pregnant women in nearby areas in south Benin (Ouidah and Comé).

## Methods

### Ethical considerations

The three studies were approved by the Ethics Committees of the Research Institute for Development in France, and of the Faculty of Medicine of Cotonou in Benin. Before each inclusion, written informed consent was obtained from all participants.

### Study sites and population

The study sites and populations have been described elsewhere [6-8]. Briefly, the three studies were conducted in three rural or semi-rural areas located within a 50 km radius in south Benin (Ouidah, Come, Allada). In the whole area, malaria is perennial and *Plasmodium falciparum* is the commonest species. There are two high transmission peaks from April to July and October to November. The study population was composed of HIV-negative pregnant women who attended one of the nine study maternity clinics (three in each site) during the period of the studies.

### Ouidah study

This study is a randomized controlled clinical trial, which took place from 2005 to 2008 in Ouidah, a 35,000-inhabitant semi-rural area located 40 km west of Cotonou, in three maternity clinics (Kindji, Kpassé, Hopital de Zone). It compared the efficacy of sulphadoxine-pyrimethamine SP (1,500/75 mg per dose) *vs* mefloquine (15 mg/kg per dose) given for IPTp on birth weight. The first IPTp dose was administered between 16 and 28 weeks, and the second dose after 30 weeks of gestation, with at least one month from the first dose intake [6].

### STOPPAM study (strategy to prevent pregnancy-associated malaria)

STOPPAM is a cohort study of pregnant women, set up in three maternity clinics (Comé central, Ouedeme-Pedah, Akodeha) in the district of Comé, with a population of 58,396 inhabitants. The district of Comé is a rural area located 70 km west of Cotonou. STOPPAM aimed to investigate the development of immune response to malaria in pregnancy. This study was carried out between 2008 and 2010. Two doses of SP-IPTp were administered according to the national guidelines, the first dose of IPTp being administered between 16 and 24 weeks of gestation. Women were followed up monthly from inclusion to delivery [7].

### APEC study (anaemia in pregnancy: etiology and consequences)

APEC is an observational cohort study nested in MiPPAD (Malaria in Pregnancy Preventive Alternative Drugs), a randomized trial comparing the efficacy of IPTp with SP (1,500/75 mg per dose) and mefloquine (15 mg/kg per dose). APEC took place in three maternity clinics in the district of Allada (Allada, Attogon, Sékou), between 2010 and 2012. The district of Allada is a semi-rural area of 91,778 inhabitants located 50 km north of Cotonou. As in the Ouidah study, the first dose of IPTp was administered between 16 and 28 weeks of gestation [8].

In the three studies, women were encouraged to consult in case of symptoms any time between scheduled ANVs. Women were systematically given supplements of oral ferrous sulfate (200 mg per day) and folic acid (5 mg per day) for home treatment, according to the guidelines of the Beninese Ministry of Health (MoH) in APEC and STOPPAM studies. In the Ouidah study, the daily dose of ferrous sulphate was 400 mg and folic acid was unchanged (5 mg per day). In the three studies, iron and folic acid prescription was renewed if necessary. Theoretically, women were supposed to continue the supplementation until three months after delivery. In cases of Hb concentration below 110 g/L, women were treated according to the severity of anaemia, i e, 200 mg of oral ferrous sulphate and 5 mg of oral folic acid twice a day for mild and mode anaemia, and referred to the tertiary hospital of the district in case of severe anaemia (Hb <70 g/L). In the latter case, 500 ml of whole blood were transfused. The total amount of blood transfusion was raised to 1,500 ml if necessary, according to the guidelines of the Beninese MoH.

### Socio-demographic and clinical data collection

At inclusion (ANV1), socio-demographic data such as age, area of residence, level of education were recorded. Women were clinically examined and parity, gestational age, weight and height were recorded. Weights were measured to the nearest 0.1 kg by using an electronic scale (Seca Corp., Hanover, MD) and heights to the nearest 0.1 cm by using a bodymeter device (Seca®206 Bodymeter; Seca Corp.). Weights and heights were measured by two nurses, and the mean of the two measurements was calculated for all participants. Body mass index (BMI) was defined as the women’s weight at enrolment (kg)/height squared (m^2^). Medical history, including the history of previous pregnancies, notation of any chronic disease (as high blood pressure, diabetes, or asthma) was also recorded.

At the time of the second antenatal visit (ANV2), at delivery and during the unscheduled visits, gestational age, weight and height were measured. Gestational age was estimated using the date of the last menstrual period and/or by measuring the fundal height.

### Blood sample collection

At ANV1, ANV2 and at delivery, venous blood was collected from each participant to determine Hb concentrations and to perform thick blood smears in search of peripheral malaria. At delivery, a placental blood smear was also performed to look for placental malaria. In the APEC study, blood was collected into dry tubes to determine women’s serum ferritin and CRP concentrations. Serum ferritin and C-reactive protein (CRP) concentrations were used to evaluate women’s iron stores. Iron deficiency was defined as serum ferritin < 12 μg / L or as serum ferritin between 12 and 70 μg/L in the context of inflammation defined as a positive CRP, i.e., CRP concentration > 5 mg/mL.

### Laboratory tests

The laboratory methods have been described elsewhere [6-8]. Haemoglobin concentration was measured with either with a haematology analyser (Erma Laboratory, Japan) or Hemo_Control® (EKF Diagnostics, Germany) device.

In the Ouidah randomized trial and STOPPAM, blood smears were performed using the standard method. Thick blood smears were stained with Giemsa and read at x100 oil immersion. Smears were considered negative if no asexual stage of malaria parasite was detected after counting 500 leucocytes. Malaria parasites were counted against 200 leucocytes [9].

In APEC, the Lambaréné technique was used. It consists of spreading 10 μL of blood on a slide’s rectangular area of 1.8 cm^2^ (1.8 cm x 1 cm). The slide is then stained with Giemsa and read at x100 oil immersion. A multiplication factor is applied to the average parasitaemia/field to get a number of parasites/μL. The Lambaréné method detection threshold has been estimated to five parasites/μL [10]. Serum ferritin was measured by using an AxSym Immuno-Assay Analyzer (Abbott Laboratories, Abbott Park, IL) with 500 mL of serum. The concentrations of serum CRP were determined by using a rapid slide test (CRP Latex; Cypress Diagnostics Inc., Campbellville, Ontario, Canada).

### Statistical analysis

Data were analysed with Stata version 11 for Windows (Stata Corp, College Station, TX, USA).

### Definition of outcome variables

Maternal Hb status was considered first as a continuous variable, and then as a categorical variable. Anaemia was defined as Hb below 110 g/L. Severe, mode and mild anaemia were defined as Hb concentrations less than 70 g/L, between 70 and 99 g/L and between 100 g/L and 109 g/L, respectively [11].

### Comparison of the three studies

Baseline characteristics of the women and the outcomes (maternal Hb concentrations and maternal anaemia at each blood assessment) were compared between the three studies. Other anaemia risk factors such as malaria, placental malaria and the timing of IPTp intakes were also compared. Proportions and means were compared using Chi2 test and Student’s test.

### Effects of gravidity on maternal haemoglobin concentrations and maternal anaemia

To determine the impact of gravidity on maternal Hb status, analyses were first separately performed on the data from each study. Afterwards, the data from the three studies were pooled to perform a logistic or linear regression depending on the outcome (binary or continuous).

To account for the timing of anti-malarial interventions, two variables measuring the intervals between IPTp1 and IPTp2, and IPTp2 and the end of the pregnancy were created. All variables with a P value less than 0.2 were considered as covariates for the multivariate linear or logistic regressions. The statistical significance of final models was set to P <0.05.

## Results

### Comparison of women’s general characteristics between the three studies

Three thousand, five hundred and ninety one (3,591) women were included in this analysis. One thousand, six hundred and one (1601) women were from the Ouidah trial, 985 women from STOPPAM and 1,005 women from APEC. Haemoglobin concentrations were assessed in 98% (72/3591) of the women at ANV1 and 89.4% (335/3164) at delivery.

The general characteristics of women and the outcomes of the three studies are presented in Table 1. The proportion of women who received education was higher in Ouidah than in STOPPAM and APEC (P <0.001). The proportions of women less than 21 years old (median age) did not differ between the three studies (P = 0.22), but there were more primigravidae in Ouidah than in STOPPAM and APEC (P <0.001). The overall nutritional status of the mothers, assessed by the body mass index (BMI) on inclusion did not differ significantly between the three studies (P = 0.07), but there was a tendency for a lower BMI in Ouidah. Placental malaria was more common in STOPPAM and APEC than in Ouidah (11.2%, 9.2% and 3.0% respectively).

**Table 1:** Comparison of women’s general characteristics between the three studies

**Factors**	**Ouidah study n = 1601**	**STOPPAM n = 985**	**APEC n = 1005**	***P value***
Area	Semi-rural	Rural	Semi-rural	
Education of the woman *(%)*				
None	44.5	56.3	66.6	*< 0.001*
Some	55.5	43.7	33.4	
Age *(%)*				
< 21	24.7	22.3	25.6	*0.22*
≥ 21	75.3	77.7	74.4	
Gravidity *(%)*				
Primigravidae	26.9	18.7	18.9	*< 0.001*
Multigravidae	73.1	81.3	81.1	
BMI at inclusion (kg /m^2^) *( % )*				
<20	81.0	78.1	77.7	*0.07*
≥20	19.0	21.9	22.3	
Interval between ANV1 and ANV2 (weeks) *mean (SD)*	8.6 (3.2)	5.1 (1.5)	6.4 (1.8)	*0.0001*
Interval between ANV2 and delivery (weeks) *mean (SD)*	8.6 (3.3)	13.9 (3.9)	12.2 (5.4)	*0.0001*
Gestational age at ANV1 (weeks) *mean (SD)*	24.2 (2.8)	20.6 (3.1)	22.1 (4.1)	*0.0001*
Gestational age at delivery (weeks) *mean (SD)*	39.6 (1.9)	39.4 (2.7)	39.3 (3.2)	*0.15*
Placental malaria *(%)*				
Positive	3.0	11.4	9.2	*< 0.001*
Negative	97.0	88.6	90.8	
Severe anaemia at ANV1 *(%)*				
Yes	1.4	0.5	0.7	*0.05*
No	98.6	99.5	99.3	
Severe anaemia at delivery *(%)*				
Yes	1.0	1.4	0.7	*0.4*
No	99.0	98.6	99.3	
Haemoglobin at ANV1 (g / L) *mean (SD)*	104.1 (14.0)	102.0 (11.5)	103.2 (12.37)	*0.0004*
Haemoglobin at ANV2 (g / L) *mean (SD)*	105.2 (13.4)	102.1 (10.5)	105.1(10.8)	*0.0001*
Haemoglobin at delivery (g / L) *mean (SD)*	113.7 (16.3)	110.0 (14.0)	111.5 (14.4)	*0.0001*
Number of antenatal visits *mean (SD)*	3.1 (0.7)	3.7 (1.3)	3.5 (1.0)	*0.0001*

SD: Standard deviation; BMI: Body Mass Index; ANV: Antenatal visit.

On average, women were given first and second doses of IPTp earlier in STOPPAM and APEC than in Ouidah (P = 0.001), but there was no significant difference between gestational ages at delivery (P = 0.15). The time intervals between IPTp intake was highest in Ouidah whereas the interval between IPTp2 and delivery was less in Ouidah than in STOPPAM and APEC. Mean Hb was higher at each blood assessment in Ouidah than in STOPPAM and APEC, and in the three studies there was a marked tendency to an increase in Hb concentrations from ANV1 to ANV2 and delivery, as described previously for APEC and the Ouidah trial [1,12]. For these reasons, we decided to include the factor “study” as an adjustment covariate in the multivariate analyses.

Twenty point five percent (20.5%) of the women had inflammation (CRP > 5 ml / ml) at ANV1 and 34.3% at delivery. The geometric means of malaria parasite density were 601.8 (CI95%: [544.6-735.1]) at ANV1 and 3294.5 (CI95%: [2208.3-4447.1]) at delivery.

### Relationship between maternal haemoglobin status and gravidity throughout pregnancy

When considering studies separately, there was an overall suppress differences between primigravidae and multigravidae at each visit that did not reach significance. After pooling data from the three studies, both univariate and multivariate analyses showed a constant association between Hb concentrations and gravidity in the three periods of Hb assessment (Table 2). Mean Hb concentration was significantly lower in primigravidae than in multigravidae at ANV1 (p < 0.001). Afterwards, it increased significantly in primigravidae only, with a tendency to reversal of the difference between primigravidae and multigravidae, which was confirmed at delivery (p < 0.001), and an overall increase in all women.

**Table 2:** Relationship between mean haemoglobin (Hb) and gravidity throughout pregnancy in south Benin

**Gravidity**	**Hb (g / L)**	Crude difference* (g / L)			Adjusted difference*^§^**(g / L)**		
		**Mean**	**CI95%**	***P value***	**Mean**	**CI95%**	***P value***
**ANV1**							
Primigravidae *(n = 786)*	100.9	−3.1	[-4.1, -2.0]	*< 0.0001*	−2.4	[-3.4, -1.4]	*< 0.001*
Multigravidae *(n = 2733)*	104.0						
**ANV2**							
Primigravidae *(n = 721)*	105.0	0.8	[-0.1, 1.9]	*0.09*	1.0	[0.004, 2.0]	*0.049*
Multigravidae *(n = 2520)*	104.2						
**Delivery**							
Primigravidae *(n =588)*	113.8	2.1	[0.7, 3.4]	*0.004*	2.8	[1.3, 4.2]	*< 0.001*
Multigravidae *(n =2241)*	111.7						

*Reference class is multigravidae.

^**§**^Adjusted for malaria on the visit, BMI, number of ANVs, intervals between ANV1 and ANV2 and between ANV2 and delivery, schooling, study and gestational age.

The following covariates were associated to lower Hb levels at ANV1: gestational age more than or equal to 16 weeks (P < 0.001), malaria (P < 0.001), low BMI (P = 0.001), study (STOPPAM and APEC studies *vs* Ouidah study, P < 0.001). At ANV2, malaria (P < 0.001), low BMI (P < 0.001), study (STOPPAM and APEC studies *vs* Ouidah study, P < 0.001) were associated to lower Hb levels. At delivery, gestational age higher than or equal to 37 weeks (P = 0.005) and more than four ANVs during the follow-up (P = 0.003) were associated with a higher Hb concentration, whereas malaria (P < 0.001) and low BMI (P = 0.012) were related to lower Hb levels.

When maternal Hb was considered as a categorical variable (anaemia or no anaemia), primigravidity remained associated with a better Hb status at delivery (aOR = 0.7, *P = 0.003*) (Table 3). The following covariates were related to an increased risk of maternal anaemia on ANV1: gestational age more than 16 weeks (P = 0.004), age below 21 years (P = 0.002), study (STOPPAM and APEC *vs* Ouidah study, P < 0.001), malaria (P < 0.001) and low BMI (P < 0.001). On ANV2, only STOPPAM study (P < 0.001), malaria (P = 0.001) and low BMI (P = 0.01) were still associated with a higher risk for maternal anaemia. At delivery, a high number of ANVs (more than four) (P = 0.001) was associated with a decreased risk for maternal anaemia, whereas malaria (P < 0.001) was related to a higher risk of maternal anaemia.

**Table 3:** Relationship between risk for maternal anaemia (Hb < 110 g/L) and gravidity throughout pregnancy in south Benin

**Gravidity**	**% of anaemia**	Crude Odds ratio*			Adjusted Odds ratio*^§^		
		**OR**	**CI95%**	***P value***	**OR**	**CI95%**	***P value***
**ANV1**							
Primigravidae *(n = 786)*	73.9	1.4	[1.1, 1.6]	*0.001*	1.1	[0.9, 1.4]	*0.28*
Multigravidae *(n = 2733)*	68.0						
**ANV2**							
Primigravidae *(n = 721)*	66.0	0.9	[0.8, 1.1]	*0.44*			
Multigravidae *(n = 2520)*	67.5						
**Delivery**							
Primigravidae *(n = 588)*	36.9	0.8	[0.7, 0.9]	*0.02*	0.7	[0.6, 0.9]	*0.003*
Multigravidae *(n = 2241)*	42.4						

*Reference class is multigravidae.

^**§**^Adjusted for malaria on the visit, BMI, number of ANVs, intervals between ANV1 and ANV2 and between ANV2 and delivery, schooling, study and gestational age.

### Relationship between gravidity, malaria infection and iron deficiency throughout pregnancy

The relationships between the two main aetiologies of anaemia, malaria and iron deficiency, and gravidity are presented in Table 4. On ANV1 and delivery, malaria was more frequent in primigravidae, but the overall decrease of malaria between ANV1 and delivery was more important in primigravidae compared with multigravidae (more than 50% of reduction in primigravidae *vs* less than 38% in multigravidae). Iron deficiency was assessed only in the APEC study. At ANV1, ANV2 and delivery, iron deficiency was more common in multigravidae, but the difference was only significant at ANV1 and ANV2.

**Table 4:** Relationship between gravidity, malaria infections and iron deficiency throughout pregnancy in south Benin, univariate analyses

**Gravidity**	Malaria infections*			**Iron deficiency**^**§**^		
**n**		**%**	***P value***	**n**	**%**	***P value***
**ANV1**						
Primigravidae	791	20.2	*< 0.001*	190	23.7	*0.002*
Multigravidae	2751	9.6		815	35.6	
**ANV2**						
Primigravidae	727	3.8	*0.44*	180	26.7	*0.003*
Multigravidae	2547	3.3		765	38.4	
**Delivery**						
Primigravidae	617	9.1	*0.005*	155	27.7	*0.4*
Multigravidae	2328	6.0		701	31.2	

*Data from the three studies.

^§^Data from APEC only (Iron status was only assessed in APEC study).

## Discussion

This study showed that if primigravidae were the most vulnerable group to anaemia at the beginning of pregnancy, they rapidly increased their Hb to reach higher levels than multigravidae at delivery. Protective interventions against malaria (i e, IPTp) appeared to play a major role in this process, which was demonstrated in three studies conducted in different areas at different times in southern Benin.

Although the women come from three different studies, they nevertheless share common factors. They all originated from the same region of Benin, with the same climatic pattern, and potentially similar malaria transmission in all sites [13]. They were all included before 28 weeks of gestation and they had the same average gestational age at delivery. Moreover, the women did not differ in the distribution of baseline characteristics such as age, body mass index at inclusion, which are prone to influence Hb concentrations [8]. However, although all women received two doses of IPTp with a minimal one-month interval between intakes, the study designs were different: STOPPAM being an observational study and Ouidah and APEC controlled randomized trials. The timing of IPTp also differed between the three studies, the last intake being on average one month closer to delivery in Ouidah compared with the two other studies and probably related to a lower prevalence of placental infection, as stressed by Huynh *et al*[14]. STOPPAM study and APEC trial were located in more rural settings than Ouidah. Finally, investigations were held at different times and one can assume that, in particular, resistance of malaria parasites to SP may have progressed from 2005 until now.

The analyses of data from each of the three studies separately showed that primigravidae who were initially at higher risk of anaemia at inclusion (ANV1), increased progressively Hb concentration and became, although not significantly, at lower risk of anaemia at delivery compared with multigravidae. Pooling all data from the three studies increased the sample size and thus the power of the analysis, demonstrating the importance of gravidity as a determinant of maternal anaemia, even after adjusting on the characteristics of each population. In addition, taking into account gestational age in the multivariate analyses minimized the effect of potential confounders such as the gestational plasma volume changes that might as well have played a role in the parity-related difference.

Primigravidae had a lower mean Hb and an increased risk for anaemia in early pregnancy compared to multigravidae, prior to the administration of IPTp. Furthermore, an overall decrease in the proportion of malaria infections after women were given IPTp has been shown [1,15-17]. In the study, more than 20% of primigravidae were infected by malarial parasites at inclusion, whereas only less than 10% of them were malaria positive at delivery. At the same time, the proportion of malaria-infected multigravidae decreased from 10% to 6%. The decline in the proportion of malaria infections in primigravidae also coincides with the increase of Hb concentrations on ANV2 and delivery. Such an increase of Hb concentrations in primigravidae may then be explained by the reduction of the proportion of malaria-related anaemia by IPTp in this group. These results are in agreement with a large meta-analysis of Hb parity differences comparing malarious and non malarious areas [18]. In addition, the demonstration of a better efficacy of anti-malarial drugs in primigravidae for the prevention of anaemia had been made in the 1990s, when chloroquine chemoprophylaxis was still used [19]. Since the implementation of IPTp with SP, two observational studies led to conflicting results [20,21]. Rogerson *et al* showed in Malawian women a decrease of maternal anaemia only in primigravidae [20], whereas Hommerich *et al* showed a decrease of anaemia in only Ghanaian multigravidae [21]. Finally, a Cochrane review of the effects of drugs to prevent malaria-related illnesses in pregnant women concluded that IPTp was effective to reduce the risk for peripheral and placental malaria and maternal anaemia, especially in primi and secundigravidae [22].

In spite of IPTp and iron supplementation, multigravidae increased Hb levels, but not in the same proportion as primigravidae, and consequently were at higher risk of anaemia at delivery. Indeed, in the APEC study, the proportion of women presenting with iron deficiency was higher in multigravidae than in primigravidae at each blood assessment. As previously described [1], there is a great demand for iron during pregnancy, as shown by the drop in Hb levels in the second trimester of gestation. Consequently, the effect of iron supplementation may be masked and even be insufficient to cover the needs of the mother and the foetus. In agreement with this hypothesis, Hernandez-Martinez *et al* showed in Spanish, well-nourished, pregnant women (including primi and multigravidae) who received an iron supplementation (40-60 mg/day) that iron deficiency increased importantly as pregnancy progressed (from 8% at inclusion to 68% at delivery) [23]. A similar trend was not observed in APEC. On the contrary, iron deficiency seemed to decrease in multigravidae (from 35% at ANV1 to 31% at delivery), suggesting that the effect of iron supplementation might be better than expected. In this study, the difference between primigravidae and multigravidae may be a consequence of an increase in the prevalence of iron deficiency, due to cumulative iron requirements of successive pregnancies. Additionally, closely spaced pregnancies that are frequently observed in sub-Saharan Africa may exacerbate this phenomenon, as birth intervals have been found to affect women’s Hb concentrations, with short intervals being a risk factor for anaemia [24].

The lack of longitudinal data for pregnant women with the same definition of iron deficiency makes comparisons of prevalence of iron deficiency difficult among studies from sub-Saharan Africa. Nevertheless, in Kenyan pregnant women of less than 24 weeks’ gestational age, results close to the findings of this study were found by Alusala *et al*[25]. The authors showed that more than 40% of multigravidae were iron deficient, defined as serum ferritin concentrations below 12 μg/L *vs* less than 19% of primigravidae. This is in agreement with the results of APEC (36% and 24% in the two groups).

## Conclusion

In the context of IPTp, primigravidae were shown to have a progressive increase in Hb concentration throughout pregnancy. The effect of IPTp on anaemia in multigravidae women was less marked, as they are less susceptible to malaria and nutritional deficiencies seem to be the main causes of anaemia in this group. There is a need to reinforce malaria prevention strategies in both groups, and to undertake additional measures focusing on the reduction of micronutrient deficiencies in multigravidae. For instance, women should be encouraged to take iron supplements from the first pregnancy until menopause, even at lower doses during the interval between pregnancies, to reduce iron side effects and increase compliance. This strategy is relatively easy to implement as the target population may be identified at the first pregnancy.

## Competing interests

The authors declare that they have no competing interests.

## Authors’ contributions

SO participated to conceive APEC study, participated in its design and coordination, performed statistical analyses and drafted and finalized the manuscript. FBL participated in statistical analyses and the finalization of the manuscript. VB participated to conceive Ouidah study, participated in its design and coordination, participated to the statistical analyses of the manuscript. BTH participated to implement and coordinate STOPPAM study and the finalization of the manuscript. GKK participated to implement APEC study, in the statistical analyses and the finalization of the manuscript. MMKA participated to the implementation and coordination of APEC study. NF participated to conceive, implement and coordinate STOPPAM study and the finalization of the manuscript. AM participated to conceive, implement and coordinate STOPPAM study and the finalization of the manuscript. PD participated to conceive and coordinate STOPPAM study and the finalization of the manuscript. MC participated to conceive and coordinate the three studies (Ouidah, STOPPAM, APEC), in the statistical analyses and the finalization of the manuscript. All authors read and approved the final manuscript.
